# Identification of Two Novel Compound Heterozygous PTPRQ Mutations Associated with Autosomal Recessive Hearing Loss in a Chinese Family

**DOI:** 10.1371/journal.pone.0124757

**Published:** 2015-04-28

**Authors:** Xue Gao, Yu Su, Yu-Lan Chen, Ming-Yu Han, Yong-Yi Yuan, Jin-Cao Xu, Feng Xin, Mei-Guang Zhang, Sha-Sha Huang, Guo-Jian Wang, Dong-Yang Kang, Li-Ping Guan, Jian-Guo Zhang, Pu Dai

**Affiliations:** 1 Department of Otorhinolaryngology, Head and Neck Surgery, PLA General Hospital, Beijing, P. R. China; 2 Department of Otolaryngology, Hainan Branch of PLA General Hospital, Sanya, P. R. China; 3 Department of Otorhinolaryngology, the Second Artillery General Hospital, Beijing, P. R. China; 4 BGI–Shenzhen, Shenzhen, 518083, China; Universitat Pompeu Fabra, SPAIN

## Abstract

Mutations in *PTPRQ* are associated with deafness in humans due to defects of stereocilia in hair cells. Using whole exome sequencing, we identified responsible gene of family 1572 with autosomal recessively non-syndromic hearing loss (ARNSHL). We also used DNA from 74 familial patients with ARNSHL and 656 ethnically matched control chromosomes to perform extended variant analysis. We identified two novel compound heterozygous missense mutations, c. 3125 A>G p.D1042G (maternal allele) and c.5981 A>G p.E1994G (paternal allele), in the *PTPRQ* gene, as the cause of recessively inherited sensorineural hearing loss in family 1572. Both variants co-segregated with hearing loss phenotype in family 1572, but were absent in 74 familial patients. Heterozygosity for c. 3125 A>G was identified in two samples from unaffected Chinese individuals (656 chromosomes). Therefore, the hearing loss in this family was caused by two novel compound heterozygous mutations in *PTPRQ*.

## Introduction

Hearing loss is very common sensory disorders in humans. Autosomal recessive non-syndromic hearing loss (ARNSHL) accounts for about 80% of hereditary hearing loss [1, 2]. To date, 55 genes and more than 100 genetic loci have been implicated in ARNSHL (http://hereditaryhearingloss.org).

Given the genetic heterogeneity of ARNSHL, the deafness genes encode a large variety of proteins with different functions, including gene regulation, fluid homeostasis, synaptic transmission, and hair cell bundle morphology and development [3, 4]. Defect in any part of this complex chain can lead to hearing impairment. However, despite previous intensive linkage analysis and candidate gene screening, a large proportion of ARNSHL cases remain genetically unexplained.

Researchers have recently identified more than 10 non-syndromic deafness genes and several dozens of novel causative mutations using targeted genomic enrichment and Whole Exome Sequencing (WES), including *TPRN*, *GPSM2*, *CEACAM16*, *SMPX*, *HSD17B4*, *HARS2*, *MASP1*, *DNMT1*, *TSPEAR*, *CRXCR2*, etc [5–14].

*PTPRQ* is located at the DFNB84 locus on chromosome 12q21.2 and consists of 45 exons comprising 234,677 base pairs (bp). The protein encoded by this gene, PTPRQ, is a protein tyrosine phosphatase receptor type Q composed of 2,299 amino acids, and plays an important role in the formation of stereocilia in hair cells of the inner ear. Loss of *Ptprq* leads to shaft-connector malformation of hair bundle in the mammalian cochlea [15]. It has been suggested that Ptprq may play multiple roles in hair cells, which include establishing the membrane at the base of the stereocilia, regulating actin dynamics in stereocilia, and participate in tethering the stereocilia membrane to the cytoskeleton [16].

Mutations within this gene at the DFNB84 loci have been reported to cause non-syndromic autosomal recessive hearing loss [17]. To date, three recessive mutations in *PTPRQ* have been shown to be associated with ARNSHL in three families from Dutch, Moroccan and Palestinian descent (Table 1). Nearly all reported recessive cases have shown a similar phenotype characterized by progressive moderate-to-profound hearing loss and vestibular dysfunction [17]. However, mutations in the *PTPRQ* gene have not to date been reported in Chinese populations.

**Table 1:** Overview of all *PTPRQ* mutations identified to date.

Origin	MutationDNA Protein		Exon	Type of variant	Inheritance pattern	Reference
Palestinian	c.1285C>T	p.Q429X	9	Nonsense	AR	[18]
Dutch	c.1491T>A	p.Y497X	10	Nonsense	AR	[17]
Moroccan	c.1369A>G	p.A457G	10	Missense	AR	[17]
China	c.3125A>G	p.D1042G	20	Missense	AR	Present study
China	c.5981A>G	p.E1994G	37	Missense	AR	Present study

AR, Autosomal Recessive

Here, we report a family with two siblings affected by congenital sensorineural hearing loss. Mutations within the *GJB2* and *SLC26A4* genes were previously excluded as causative genes. WES was performed in two affected siblings and two unaffected parents, followed by polymerase chain reaction (PCR) and Sanger validation. These procedures identified two compound heterozygous disease-segregating mutations, c. 3125 A>G (p.D1042G) and c.5981 A>G (p.E1994G), within the *PTPRQ* gene. In addition, DNA samples from 74 patients from ARNSHL families and 328 unaffected individuals (656 chromosomes) were also analyzed.

## Methods and Materials

### Clinical data

Family 1572 is a two-generation Chinese family with autosomal recessive prelingual non-syndromic sensorineural hearing loss. To screen for candidate mutations, 328 ethnically matched controls (656 chromosomes) and 74 affected DNA samples from the Department of Otolaryngology, Head and Neck Surgery, Chinese PLA General Hospital were analyzed. The 74 affected patients originated from 74 families presenting with ARNSHL, and in whom mutations within *GJB2* and *SLC26A4* genes had been excluded previously. Fully informed written consent was obtained from all subjects or, in the case of children, from their guardians. The study was approved by the Chinese PLA General Hospital Research Ethics Committee. Medical histories, otoscopy, physical examination, immittance testing and pure tone audiometric examination of family 1572 members were performed as described in detail previously [19]. The parents reported no history of stillbirth or miscarriage. Computed tomography (CT) scan of the temporal bone was also performed. The diagnosis of profound sensorineural hearing impairment was made according to the WHO criteria based on audiometric examination. Vestibular function was evaluated by tandem gait and Romberg testing.

### DNA preparation

All genomic DNA was extracted from peripheral blood using a blood DNA extraction kit according to the manufacturer’s instructions (TianGen, Beijing, China).

### Whole-exome capture and sequencing

Whole-exome capture and sequencing have been described in detail previously [19]. In brief, genomic DNA was captured using the NimblegenSeqCap EZ Library (44 Mb for I1, I2, II:1, II:2). Each captured library was then loaded onto the Illumina Hiseq2000 platform. Raw image files were processed using the Illumina base calling software 1.7. Sequence read data of four subjects in family 1572 has been deposited into Sequence Read Archive (http://www.ncbi.nlm.nih.gov/sra website; accession number SRP050281).

Read mapping, variant detection, filtering, and annotation have been described in detail previously [19]. According to autosomal-recessive pattern of inheritance, only variants that were homozygous or compound heterozygous in the two affected siblings and heterozygous in their parents were selected as candidates.

### Mutation validation

After filtering against multiple databases, Sanger sequencing was used to determine whether any of the potential novel mutations within known causative genes of ARNSHL co-segregated with the disease phenotype in this family. Direct PCR products were sequenced as described in detail previously [19].

### Mutation analysis

Mutation segregation was performed in all family members. Additionally, 328 negative samples and 74 familial patients were also screened for the mutations by direct sequencing. Genotyping for c. 3125 A>G and c.5981 A>G was performed by PCR (primer sequences are available upon request) and detected by bidirectional sequencing of the amplified fragments using an automated DNA sequencer (ABI 3100; Applied Biosystems). Nucleotide alteration(s) were identified by sequence alignment with the *PTPRQ* GenBank sequence (NM_001145026.1) using the Genetool software.

### Multiple sequence alignment

Multiple sequence alignment was performed according to a Homologene program with default settings and the following sequences: NP_001138498.1 (*Homo sapiens*), XP_001085761.2 (*Macaca mulatta*), XP_001151129.2 (*Pan troglodytes*), NP_001179484.1 (*Bos taurus*), XP_539698.3 (*Canis lupus*), NP_001074901.1 (*Mus musculus*), NP_075214.1 (*Rattus norvegicus*), XP_001235338.2 (*Gallus gallus*), and XP_683017.4 (*Danio rerio*).

### Model building and structural-based analysis

Three-dimensional (3D) modeling of the human wild-type, p.D1042G mutation and p.E1994G mutation were performed using SWISS-MODEL, an automated homology modeling program (http://swissmodel.expasy.org/workspace/). This study used the automatic modeling approach to apply the complete protein sequence of human PTPRQ, including its 2,299 amino acids and its mutation, which are available in the NCBI GenBank (NP_001138498.1) in FASTA format. Data obtained by the homology models were visualized using Swiss-PdbViewer 4.1.

## Results

### Clinical presentation of family 1572

**Fig 1:**
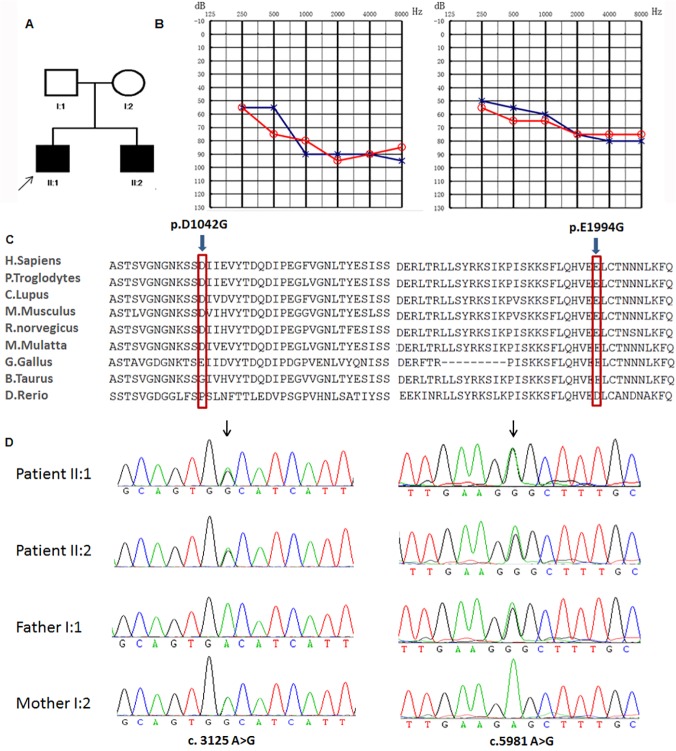
Combined figure. A. Pedigree of family 1572 with ARNSHL. Affected subjects are denoted in black. Arrow indicates the proband, B. Audiogram of affected subjects shows bilateral profound hearing loss ranging from severe to profound from subjects II:1 and II:2 (red, right ear; blue, left ear). C. Conservation analysis. Protein alignment shows conservation of residues TMC1 D1042 and E1994 across nine species. These two mutations occur at an evolutionarily conserved amino acid (red). D. Electropherogram analysis of PTPRQ in family 1572 shows that compound heterozygous mutations (c.3125 A>G and c.5981 A>G) co-segregate with the phenotype.

Family 1572 includes two affected siblings (22 and 6 years old) and two unaffected parents (Fig 1A). Audiograms of the affected siblings revealed that the hearing loss was bilateral and moderate-to-profound with prelingual onset (Fig 1B). Hearing loss in individual II:1 shows slight progression with annual threshold deteriorations about 1~2dB in average at the frequencies 0.25, 0.5kHz. II:2 did not pass the hearing screening at the age of 42 days. In early childhood, the hearing loss in this family must have been less severe that speech development was normal in two patients. Immittance testing demonstrated normal and bone conduction values equal to the air conduction measurements, suggesting a sensorineural hearing impairment. The affected individuals did not show delays in gross motor development, nor did they have balance problems, vertigo, dizziness, or spontaneous or positional nystagmus. Tandem walking was normal, and the Romberg test was negative. A CT scan of the temporal bone in the proband excluded inner-ear malformations. Physical examination of all family members revealed no signs of systemic illness or dysmorphic features. The remaining examination results were completely normal. To exclude mutations in the genes known to be associated with hereditary hearing loss, *SLC26A4* and *GJB2* were sequenced in the proband (patient II:1, Fig 1A). However, no mutations were found in these genes, facilitating us to sequence the whole exomes of these two patients.

### Whole-exome sequencing (WES)

WES was applied to the two affected siblings and two unaffected parents: I:1, I:2, II:1, and II:2. The reads were aligned with the human genome reference sequence. On average, 6.30 billion bases of sequence were generated with mean target region depths of 73.6. Approximately 99.1% (3,253 Mb in length) of the targeted bases were covered sufficiently to pass our thresholds for calling single nucleotide polymorphisms (SNPs) and short insertions or deletions (indels). The average rates of nucleotide mismatch were 0.34%. After identification of variants, only non-synonymous variants (NS), splice acceptor and donor site mutations (SS), and frameshift coding indels were used for further analysis; these are more likely to be pathogenic than others, especially those in homozygous or compound heterozygous modes (S1 Table).

On average, 95,337 SNPs in the coding regions and 2,521 variants in introns were identified that may affect splicing (within 10 bp of the intron/exon junction) for each sample (S2 Table). Next, these variants were compared with the dbSNP135 (http://www.ncbi.nlm.nih.gov/projects/SNP/), HapMap project (ftp://ftp.ncbi.nlm.nih.gov/hapmap), 1000 Genome Project (ftp://ftp.1000genomes.ebi.ac.uk/vol1/ftp), and YH databases (http://yh.genomics.org.cn/). Potential candidate variants were identified by selecting the homozygous or compound heterozygous variants found in both affected siblings while unaffected parents contributed equally. Under the autosomal recessive mode of inheritance, 22 genes with compound heterozygous variants and two genes with homozygous variants were identified. Next, these variants were compared to reported non-syndromic hereditary hearing loss genes, then two mutations (c.3125 A>G and c.5981 A>G) were found in a previously reported deafness-related gene, *PTPRQ*.

### Mutation detection and analysis

Amino acid substitutions p.D1042G and p.E1994G were analyzed with SIFT (http://sift.bii.a-star.edu.sg/) and Polyphen (http://genetics.bwh.harvard.edu/pph2/), which predicted them to be tolerated. The c. 3125 A>G substitution occurs in exon 20 of *PTPRQ* and results in a single amino acid substitution (aspartic acid to glycine; p.D1042G) within the FNIII 11 domain. The c.5981 A>G substitution occurs in exon 37 of *PTPRQ* and results in a single amino acid substitution (glutamic acid to glycine; p.E1994G) within the topological domain. Both mutations affect the conserved residues (Fig 1C). Sanger sequencing validated the two affected siblings’ mutations and demonstrated that the two parents were heterozygous carriers of c.3125 A>G (maternal allele) and c.5981 A>G (paternal allele), showing complete co-segregation of the mutation with the phenotype (Fig 1D).

Using direct sequencing, 656 ethnically matched control chromosomes and 74 familial patients with ARNSHL were genotyped to identify the mutations. c.3125 A>G was identified (in a heterozygous state) in two individuals among the 328 ethnically matched controls (656 chromosomes), and c.5981 A>G was absent in 656 negative chromosomes. These two mutations were absent in 74 familial patients as well as the dbSNP135, HapMap project, 1000 Genome Project and YH databases. These observations suggest that the p.D1042G and p.E1994G mutations are likely to have a detrimental effect on the protein.

### Structure modeling of p.D1042G and p.E1994G

**Fig 2:**
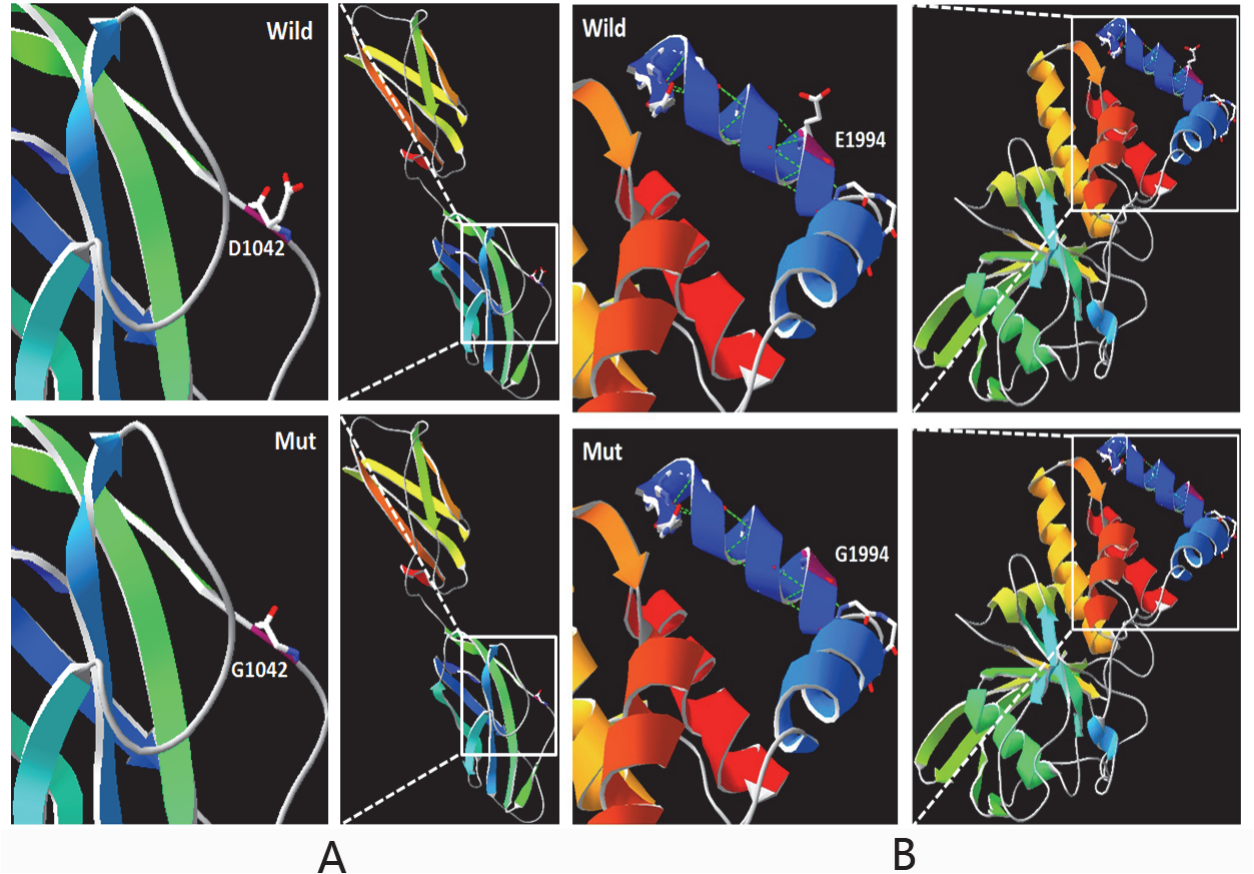
Molecular modeling of wild-type and mutant PTPRQ. A. D1042 lies within a loop between two strands. The wild-type protein has a longer side chain than the mutant protein. B. The mutant protein G1994 has a shorter side chain than the wild-type protein and is predicted to lose two hydrogen bonds (created by SWISS-MODEL and shown with PY-MOL).

The D1042G molecular model covered the target sequence of PTPRQ (residues 958–1,134), and was constructed based on the crystal structure of the axon guidance receptor (PDB ID: 4hljA). Sequence identity between the target and template was 25%. Using Swiss-Pdb Viewer 4.1, the mutation was predicted to perturb the amino acid side chain due to the substitution of aspartic acid to glycine at position 1,042. The mutant residue is also smaller than the wild-type residue (Fig 2A).

The E1994G molecular model covered the target sequence of PTPRQ (residues 1,982–2,260), and was constructed based on the crystal structure of the catalytic domain of PTPRQ (PDB ID: 4ikcA). Sequence identity between the target and template was 97.13%, which is considerably higher than the average (25%). Using Swiss-Pdb Viewer 4.1, the mutation was predicted to perturb the amino acid side chain due to the substitution of glutamic acid to glycine at position 1,994 (Fig 2B). The wild-type glutamic acid forms a salt bridge with the lysine at position 2,002. The difference in charge will likely disturb the ionic interaction made by the wild-type residue.

These data, together with the clinical presentation of the two affected siblings, clearly indicate that *PTPRQ* mutations are the cause of ARNSHL in this family.

## Discussion

We report here the use of WES approach in a small nonconsanguineous family including 2 affected individuals with ARNSHL. This approach led us to the identification of two novel compound heterozygous mutations in *PTPRQ* gene. *PTPRQ* mutations are rare causes of recessive deafness as there are only two reports worldwide. This is the first study to identify *PTPRQ* as the ARNSHL-associated gene in a Chinese population.

PTPRQ, a receptor-like phosphatase, have shown to be active against phosphatidylinositol phosphates which is involved in the cell regulation, proliferation [20]. Quantitative PCR analysis identified widespread expression in human fetal tissues, with highest expression in kidney, lung and cochlea [17]. PTPRQ belongs to the membrane protein and includes an extracellular domain that contains multiple fibronectin III repeats and a cytoplasmic domain with phosphatidylinositol phosphatase activity [21, 22].

The c. 3125 A>G substitution occurs within the extracellular FNIII 11 domain that contains a recognition sequence in a flexible loop between two strands. These extracellular domains are able to bind ligands including extracellular proteins and other molecules such as collagen and heparin, respectively, as well as ligands on the cell [23–25]. The wild-type residue is negatively charged while the mutant residue is neutral, and thus may cause a loss of interactions with other molecules or residues. The c.5981 A>G substitution occurs within the topological domain. The wild-type residue forms a salt bridge with the lysine at position 2,002. The difference in charge may disturb the ionic interaction by the wild-type residue. The charge of the wild-type residue could be lost if replaced by the mutant residue; this can cause a loss of interactions with other molecules or residues. The mutant residue is smaller, and thus may also lead to a loss of interactions. The mutation introduces a more hydrophobic residue and can result in a loss of hydrogen bonds and/or disturb folding [26]. Glycine residues are very flexible; thus, the p.D1042G and p.E1994G substitutions may affect protein stability and consequently function at these positions. Moreover, Mupro (http://mupro.proteomics.ics.uci.edu/) predicts that the mutations decrease protein stability.

In summary, our findings confirm that novel compound heterozygous mutations in *PTPRQ* are the pathogenic cause of ARNSHL in a non-consanguineous Chinese family. Identification of these two mutations in *PTPRQ* further confirms its crucial role in auditory function.

The English in this document has been checked by at least two professional editors, both native speakers of English. For a certificate, please see: http://www.textcheck.com/certificate/14r3OH

## Supporting Information

S1 Table Overview of data production using WES. (DOCX)

S2 Table Summary of SNPs for exome capture samples. (DOCX)
