# Supplementary material for: Identification of Two Novel Compound Heterozygous PTPRQ Mutations Associated with Autosomal Recessive Hearing Loss in a Chinese Family
Source: PLoS One. 2015 Apr 28;10(4):e0124757. doi: 10.1371/journal.pone.0124757 (PMC4412678; doi:10.1371/journal.pone.0124757)
Supplement: S1 Table — (DOCX) [file pone.0124757.s001.docx]

**Table S1 Overview of data production using WES**

| **Exome Capture Statistics** | **I:1** | **I:2** | **II:1** | **II:2** |
| --- | --- | --- | --- | --- |
| Target region (bp)^(1)^ | 44250712 | 44148032 | 44250712 | 44250712 |
| Raw reads | 63512248 | 84626494 | 66120990 | 65591878 |
| Raw data yield (Mb) | 5716 | 7616 | 5951 | 5903 |
| Reads mapped to genome | 57204397 | 75548490 | 59403890 | 58596963 |
| Reads mapped to target region^(2)^ | 41391703 | 55392255 | 42869194 | 42538500 |
| Data mapped to target region (Mb) | 2953.96 | 3967.86 | 3055.18 | 3033.06 |
| Mean depth of target region(X) | 66.76 | 89.88 | 69.04 | 68.54 |
| Coverage of target region (%) | 99.09 | 99.34 | 99.1 | 99.05 |
| Average read length (bp) | 89.95 | 89.95 | 89.95 | 89.95 |
| Rate of nucleotide mismatch (%) | 0.35 | 0.3 | 0.35 | 0.34 |
| Fraction of target covered >=4X (%) | 97.6 | 98.27 | 97.63 | 97.5 |
| Fraction of target covered >=10X (%) | 94.38 | 96.18 | 94.46 | 94.12 |
| Fraction of target covered >=20X (%) | 87.26 | 91.84 | 87.49 | 86.81 |
| Capture specificity (%)^(3)^ | 74.35 | 75.17 | 74.21 | 74.64 |
| Reads mapped to flanking region^(4)^ | 8148383 | 10237769 | 8569620 | 8226092 |
| Mean depth of flanking region(X) | 21.04 | 27.22 | 22 | 21.51 |
| Coverage of flanking region (%) | 96.39 | 97.37 | 96.55 | 96.14 |
| Fraction of flanking covered >=4X (%) | 83.95 | 87.97 | 84.76 | 83.33 |
| Fraction of flanking covered >=10X (%) | 60.37 | 67.63 | 61.78 | 60.12 |
| Fraction of flanking covered >=20X (%) | 36.73 | 45.22 | 38.25 | 37.18 |
| Fraction of unique mapped bases on or near target (%) | 87.98 | 88.14 | 88.01 | 88.12 |
| Duplication rate (%)^(5)^ | 6.21 | 7.89 | 6.43 | 6.99 |
| Mean depth of chrX(X) | 45.81 | 119.4 | 47.24 | 46.49 |
| Mean depth of chrY(X) | 92.86 | - | 94.72 | 94.86 |
| GC rate (%) | 44.72 | 44.94 | 44.48 | 44.25 |
| Gender test result | M | F | M | M |
