# Supplementary material for: Identification of Two Novel Compound Heterozygous PTPRQ Mutations Associated with Autosomal Recessive Hearing Loss in a Chinese Family
Source: PLoS One. 2015 Apr 28;10(4):e0124757. doi: 10.1371/journal.pone.0124757 (PMC4412678; doi:10.1371/journal.pone.0124757)
Supplement: S2 Table — (DOCX) [file pone.0124757.s002.docx]

**Table S2 Summary of SNPs for exome capture samples**

| **Categories** | **I:1** | **I:2** | **II:1** | **II:2** |
| --- | --- | --- | --- | --- |
| Number of genomic positions for calling SNPs^(1)^ | 127078147 | 126771780 | 127078147 | 127078147 |
| Number of high-confidence genotypes^(2)^ | 116269233 | 117665652 | 116542161 | 115852589 |
| Number of high-confidence genotypes in TR | 43303162 | 43390026 | 43309938 | 43287272 |
| Total number of SNPs | 93657 | 99942 | 94316 | 93434 |
| Nonsense | 116 | 129 | 126 | 121 |
| Readthrough | 45 | 49 | 48 | 42 |
| Missense | 10921 | 11321 | 10883 | 11027 |
| Splice site^(3)^ | 2484 | 2594 | 2498 | 2507 |
| 5-UTR | 2327 | 2667 | 2388 | 2338 |
| 3-UTR | 4927 | 5236 | 4936 | 4891 |
| NR_exon | 8416 | 8827 | 8349 | 8441 |
| Synonymous-coding | 5782 | 6022 | 5786 | 5818 |
| Intron | 56098 | 60322 | 56675 | 55774 |
| Intergenic | 2541 | 2775 | 2627 | 2475 |
| Hom | 39124 | 40370 | 39161 | 38450 |
| Het | 54533 | 59572 | 55155 | 54984 |
